# Phosphatase Signaling as a Therapeutic Strategy in Schizophrenia

**DOI:** 10.3390/ijms27062822

**Published:** 2026-03-20

**Authors:** Lauren E. Molony, Lutz Tautz

**Affiliations:** Center for Therapeutics Discovery, Sanford Burnham Prebys Medical Discovery Institute, La Jolla, CA 92037, USA; lmolony@sbpdiscovery.org

**Keywords:** schizophrenia, neuropsychiatric disorders, synaptic plasticity, therapies, phosphatases, inhibitors, STEP, PTP1B, calcineurin, PP1

## Abstract

Cognitive impairment in schizophrenia remains insufficiently addressed by existing treatments. Current FDA-approved therapies primarily modulate neurotransmitter systems, resulting in incomplete symptom control and substantial adverse effects. There is therefore a critical need for therapeutic strategies that more directly address the intracellular signaling mechanisms underlying synaptic dysfunction and cognitive deficits in schizophrenia. Protein phosphatases represent an essential but historically underexplored class of signaling enzymes that regulate phosphorylation-dependent control of synaptic receptor trafficking, plasticity, and neuronal circuit function. Although multiple phosphatases have been implicated in schizophrenia through genetic, post-mortem, and functional studies, their therapeutic targeting has been limited by challenges related to selectivity, cellular permeability, and pleiotropy. Here, we review the etiology of schizophrenia and limitations of current pharmacological approaches, synthesize evidence linking specific protein phosphatases to schizophrenia pathophysiology, and discuss emerging strategies, including allosteric modulation and targeted protein degradation, that may enable selective intervention in phosphatase-driven signaling pathways. We highlight the striatal-enriched tyrosine phosphatase STEP (*PTPN5*) as a case study illustrating how selective phosphatase modulation can restore synaptic signaling in schizophrenia-relevant models.

## 1. Introduction

Schizophrenia is a chronic neuropsychiatric disorder broadly characterized by severe cognitive and behavioral symptoms that negatively affect quality of life and daily functioning, leading to poor social, economic, and health outcomes. It has been recognized as a distinct mental illness for little more than a century, though descriptions of related symptoms appear in much earlier medical and philosophical texts. In the late 19th century, German psychiatrist Emil Kraepelin systematically characterized the disorder as dementia praecox, emphasizing its early onset and progressive cognitive decline, thereby distinguishing it from mood disorders [1]. In the early 20th century, Eugen Bleuler introduced the term schizophrenia, reframing the illness as a disorder of “splitting” in psychological functions rather than inevitable deterioration, and highlighting core features such as disorganized thinking, altered affect, and impaired reality testing [2]. Throughout much of the 20th century, schizophrenia was variably attributed to psychoanalytic, social, and biological causes, with treatment approaches ranging from institutionalization and psychotherapy to electroconvulsive therapy [3]. The mid-20th-century discovery of antipsychotic medications, beginning with chlorpromazine, marked a major shift toward neurobiological models centered on neurotransmitter dysfunction [4]. More recently, advances in genetics, neurodevelopment, and systems neuroscience have reshaped understanding of schizophrenia as a heterogeneous, polygenic disorder involving disrupted brain development, synaptic dysfunction, and altered neural connectivity rather than a single disease entity [5].

Presently, symptoms of schizophrenia are broadly divided into three classes: positive, negative, and cognitive. Positive symptoms refer to thoughts and behaviors that extend beyond typical functioning, such as the hallucinations, delusions, and disorganized speech and behavior that define psychosis, or loss of contact with reality. Hallucinations are false perceptions of sensory experiences, such as hearing voices that aren’t real. Delusions are irrational beliefs that do not reflect reality, such as believing one’s thoughts are being broadcast. Disorganized speech and behavior can manifest as speech that is jumbled or nonsensical, and agitated or exaggerated movements. Negative symptoms are defined as the absence of normal thoughts and behaviors, such as anhedonia (an inability to feel pleasure), avolition (a lack of willpower), and blunted affect (a lack of perceived emotion in speech and behavior). Cognitive symptoms include issues with working memory, and general executive dysfunction. Because schizophrenia is a polygenic disorder influenced by both early neurodevelopment and environmental exposures, pathophysiology and symptomology are heterogeneous across patients, making it difficult to precisely identify the etiology of schizophrenia and treat accordingly [6]. Though genetic association studies have identified multiple candidate loci and genes, no single variant or combination of genes has been found to be necessary or sufficient to cause schizophrenia. Instead, neurophysiological and neuroimaging studies have focused on characterizing abnormal network activity and gray matter deficits, particularly in the prefrontal cortex and fronto-striato-thalamic tract [7,8,9]. At the cellular level, these deficits are due to loss of synaptic connections and myelinated axons, rather than loss of neurons [10]. As such, current pharmacological treatments for schizophrenia focus on treating the symptoms rather than addressing the causes of schizophrenia.

Adverse health outcomes for schizophrenia are also high: people with schizophrenia in comparison to the general population exhibit 2.5 times increased risk for all-cause mortality and almost ten times increased risk for suicide-related mortality [11]. Unemployment rates for people with schizophrenia are similar across Europe and the United States (US) at 70–90%, contributing to the overall economic burden of schizophrenia in the US, which reaches $343.2 billion [12,13]. While current pharmacological treatments can ameliorate the more acute psychotic symptoms that impair daily functioning such as hallucinations and delusions, other schizophrenia symptoms and the side effects of these medications such as avolition, blunted affect, attention, and working memory issues continue to limit normal daily functioning [14]. In fact, meta-analyses have indicated that negative and cognitive symptoms are more strongly associated with poor quality of life (QoL) than positive symptoms. Internalized stigma and limited social support systems further exacerbate poor QoL outcomes [15]. More effective therapeutics targeting the underlying pathophysiology of schizophrenia are needed to more comprehensively address schizophrenia symptomatology and improve quality of life.

Current pharmacological strategies for schizophrenia remain dominated by antipsychotic drugs that directly or indirectly antagonize dopamine D2 receptors such as risperidone and clozapine, respectively. These drugs are effective for reducing positive symptoms but have limited efficacy for negative symptoms and cognitive impairment and are associated with substantial metabolic and cardiovascular side effects [10]. Recent advances, however, have begun to broaden the therapeutic landscape beyond dopamine blockade (Table 1). Pharmacological strategies targeting signaling proteins that contribute to the synaptic dysfunction characteristic of schizophrenia may represent a more promising therapeutic approach. Cognitive impairment in schizophrenia is increasingly attributed to disrupted regulation of synaptic plasticity, particularly alterations in the balance between long-term potentiation (LTP) and long-term depression (LTD), driven by abnormalities in glutamatergic transmission and intracellular signaling [16]. Key molecular targets include ionotropic and metabotropic glutamate receptors and their modulators, including protein kinases and phosphatases.

Protein phosphatases represent a mechanistically distinct class of signaling regulators compared with receptors or protein kinases. Whereas receptors primarily detect extracellular cues and kinases amplify signaling through phosphorylation, phosphatases act as critical integrators that dynamically constrain, redirect, and spatially refine these signals. By reversing phosphorylation events, phosphatases regulate not only the magnitude but also the timing and subcellular localization of signaling cascades, thereby shaping synaptic efficacy, receptor trafficking, and plasticity. This regulatory role is particularly relevant to neural processes underlying cognition, where stable yet adaptable synaptic modifications are required for learning and memory. Disruption of phosphorylation-dependent signaling processes has been repeatedly implicated in schizophrenia, especially in relation to cognitive impairment and negative symptoms, which are increasingly linked to deficits in cortical network function rather than isolated neurotransmitter abnormalities. Within this framework, phosphatases are positioned not merely as passive antagonists of kinases but as key determinants of synaptic and circuit-level signaling states, providing a complementary perspective to receptor- and kinase-centric models of disease.

Protein phosphatases that have been linked with schizophrenia include the serine/threonine phosphatases (STPs) PP1 (*PPP1C*) [17] and calcineurin (*PPP3C*) [18], as well as the protein tyrosine phosphatases (PTPs), PTPD1 (*PTPN21*) [19], LAR (*PTPRF*) [10], RPTPα (*PTPRA*) [20], RPTPβ/ζ (*PTPRZ*) [21], PTP1B (*PTPN1*) [22], and STEP (*PTPN5*) [23]. This review outlines the clinical features and existing therapeutics for schizophrenia, examines promising phosphatases that may modulate its pathophysiology, and synthesizes current strategies for effectively targeting phosphatases with small molecules.

**Table 1:** Current and Emerging Pharmacological Strategies for Schizophrenia and Their Limitations.

Therapy	Primary Targets	Mechanism of Action	Symptom Domain Targeted	Clinical Status	Key Limitations	Citations
Typical antipsychotics (e.g., haloperidol, chlorpromazine)	D2R (±D1R)	Broad antagonism of D2R in mesolimbic pathway; some agents engage D1R and indirectly	Positive	Approved	High EPS liability; limited efficacy for negative/cognitive symptoms	[8,24]
Atypical antipsychotics (e.g., clozapine, risperidone, olanzapine)	D2R (±D1R), 5-HT2A	Reduced D2 antagonism + 5-HT2A blockade releases dopamine in mesocortical pathways; some agents engage D1R	Positive	Approved	Metabolic side effects (weight gain, dyslipidemia); limited efficacy for cognitive symptoms	[24,25]
Partial D2 agonists (e.g., aripiprazole, brexpiprazole, lumateperone)	D2R (partial agonist) (±D1R, D3R), 5-HT receptors	Dopamine “stabilization” rather than full blockade; lumateperone engages D1R	Positive	Approved	Restlessness; Limited efficacy for negative and cognitive symptoms	[24]
KarXT (xanomeline–trospium)	M1/M4 mAChRs	Indirect modulation of dopamine circuits; M4 reduces mesolimbic dopamine; M1 enhances cortical signaling	Positive, Negative, Cognitive	Approved	Exact MOA still under investigation	[26,27]
mGlu2/3 agonists/PAMs (e.g., pomaglumetad)	Group II mGlus	Reduce presynaptic glutamate release; normalize corticolimbic E/I imbalance	Positive, Negative, Cognitive	Phase II–III	Variable efficacy	[10]
Glycine/D-serine/GlyT1 inhibitors	NMDAR co-agonist site	Enhance NMDAR function by increasing glycine availability	Negative, Cognitive	Phase II–III	Limited efficacy	[10]
KAT II inhibitors	KAT II (kynurenine aminotransferase II)	Inhibit conversion of kynurenine to kynurenic acid (KYNA), relieving endogenous inhibition of NMDARs	Cognitive	Phase I–II	Side effects remain to be fully characterized	[10]
NMDAR PAMs	NMDAR	Directly enhance receptor activity	Negative, Cognitive	Phase I	Risk of excitotoxicity	[10]
Voltage-gated sodium channel blockers (e.g., evenamide)	NaV channels	Reduce glutamate release indirectly	Positive, Negative	Open-label	Nonspecific CNS effects	[10]
LSD1 inhibitors	LSD1 (KDM1A)	Epigenetic modulation downstream of SETD1A dysfunction; alter transcriptional programs affecting synaptic plasticity	Negative, Cognitive	Phase II	Long-term safety under evaluation	[28]
cAMP/PKA pathway inhibitors	PKA signaling	Rescue network hyperactivity downstream of SETD1A mutations	Cognitive (preclinical)	Preclinical	Potential off-target effects	[29]
Kinase modulators (e.g., targeting AKT1, PTK2B/Pyk2)	AKT1, Pyk2	Modulate phosphorylation-dependent synaptic signaling and NMDAR regulation	Cognitive, Negative	Preclinical	Kinase pleiotropy complicates selectivity	[30]
Phosphatase-targeting strategies (emerging)	PP1, STEP, others	May restore balance in phosphorylation-dependent signaling, NMDAR trafficking, and synaptic strength	Cognitive, Negative (hypothesized)	Preclinical/Conceptual	Historically underexplored	[31,32]

## 2. Neurobiological Mechanisms of Current Schizophrenia Treatments

Current standard-of-care pharmacological treatments for schizophrenia are antipsychotics. Antipsychotics are commonly split into two categories: typical and atypical. Typical antipsychotics function primarily as dopaminergic antagonists, binding mainly to the D2 receptor (D2R). While their principal mechanism involves D2R blockade, some typical antipsychotics also exhibit affinity for the D1 receptor (D1R). Modulation of D1R signaling has been associated with increased phosphorylation of the GluN2B subunit of the N-methyl-D-aspartate receptor (NMDAR), which may enhance NMDAR activity [24]. Antagonism of D2R is widely considered to underlie the improvement of positive symptoms through preventing excessive dopaminergic signaling postulated to occur in the mesolimbic pathway [8]. Prefrontal dopaminergic transmission, including D1R-signaling, has been implicated in the modulation of negative and cognitive symptoms [24]. However, typical antipsychotics commonly cause adverse extra-pyramidal side effects (EPS): broad D2R blockade induces movement-related disorders such as akathisia, dystonia, tardive dyskinesia, and Parkinsonism. As a result, atypical antipsychotics were developed. Atypical antipsychotics block both serotonin and dopamine receptors but engage dopamine receptors to a lesser extent. By blocking 5-HT2 serotonin receptors, atypical antipsychotics release serotonin-mediated inhibition of dopamine receptors, which may attenuate antipsychotic-induced dopamine deficiency in the mesolimbic pathway and compensate for low dopaminergic signaling in the mesocortical pathway [24]. Though there is relief of positive symptoms and less prevalence of EPS as a result, other adverse effects, such as weight gain, hyperglycemia, and dyslipidemia, remain and negatively affect quality of life and health span [25]. Treatments for schizophrenia that avoid these adverse effects and more effectively address negative and cognitive symptoms are in great demand.

One recent advance toward clinically improved treatments for schizophrenia is the development of M1/M4 muscarinic acetylcholine receptor (mAChR) agonist xanomeline-tropsium chloride (KarXT), the first approved drug for schizophrenia that does not target D2R. KarXT has shown promise in relieving positive, negative, and cognitive symptoms of schizophrenia [33,34]. KarXT is thought to indirectly inhibit dopaminergic signaling in the mesolimbic pathway relative to the striatal pathway and restore abnormal D1R signaling in the striatum through M4 mAChR activation, perhaps relieving positive symptoms without causing EPS [26]. KarXT is also thought to increase dopamine release and restore abnormal D1R signaling via M1 mAChR activation, enhancing neuronal signaling in NMDA hypofunction states in the prefrontal cortex (PFC) and therefore remediating the cognitive symptoms of schizophrenia [27]. More studies are needed to more robustly elucidate KarXT’s mechanism of action in reducing symptoms of schizophrenia. Taken together, these proposed mechanisms suggest that KarXT may exert its therapeutic effects in part by normalizing underlying glutamatergic dysfunction, as it is thought to indirectly modulate D1R, which influences NMDAR activity [24], and enhances neuronal signaling under NMDA-hypofunction conditions in the PFC [27]. Indeed, dopamine, the primary target of traditional antipsychotics, is a modulator of both the glutamatergic and GABAergic systems in the cortex, suggesting that schizophrenia may arise from an imbalance of these neurotransmitter systems [6]. Although extensive evidence supports the NMDAR hypofunction model of schizophrenia, no effective NMDAR-directed treatments have been realized [35]. This lack of efficacious NMDAR-directed therapeutics reveals a critical gap in current treatment strategies and indicates the necessity for approaches that specifically correct underlying excitatory-inhibitory dysregulation.

Genetic studies have identified variants associated with schizophrenia that encode NMDAR, as well as other molecular players. Both genome-wide association studies (GWAS), identifying common single-nucleotide polymorphisms (SNPs), and sequencing studies, identifying rare single-nucleotide variants (SNVs), have converged on *GRIN2A*, which encodes the GluN2A subunit of NMDAR, as a risk gene for schizophrenia. Reduced channel function due to SNVs or gene expression due to SNPs in *GRIN2A* is thought to impair hippocampal synaptic plasticity and attention [7,36]. Sequencing studies have also identified *GRIA3*, which encodes an α-amino-3-hydroxy-5-methyl-4-isoxazolepropionic acid receptor (AMPAR) subunit, as a risk gene for schizophrenia, further supporting glutamatergic dysfunction as a hallmark of schizophrenia etiology. Other risk genes for schizophrenia converged upon by analysis of GWAS include *CUL9*, *FURIN*, *LINC00320*, *SNAP91*, *ZNF823* and *SP4*, which are involved in proteasomal degradation, proteolysis, glioma suppression, synaptic vesicle recycling, and transcriptional regulation in the brain, respectively [7,37,38,39]. *SETD1A*, *CUL1*, *XPO7*, *TRIO*, *CACNA1G*, *SP4*, *GRIA3*, *HERC1* and *RB1CC1*, exome-wide significant SNVs for schizophrenia, are similarly involved in transcriptional regulation and proteasomal degradation, as well as nuclear transport regulation, neuronal cytoskeleton regulation, calcium channel and autophagy function [40,41,42,43]. To date, mouse models for only *SETD1A*, *GRIA3*, *GRIN2A*, and *XPO7* SNVs demonstrated forms of altered synaptic plasticity and schizophrenia-related behavioral deficits [40,44,45,46].

While both common SNPs and rare harmful SNVs implicate overlapping molecular pathways involved in ion channel function, intracellular trafficking, and activity-dependent plasticity, no single disease-causing gene or combination of genes converging on a single druggable target has been identified yet. Thus, genetic studies primarily point to downstream signaling processes such as those involved in regulating synaptic strength and excitation–inhibition balance as critical determinants of disease pathophysiology. Current research and drug discovery efforts focus on further characterizing and targeting the downstream signaling molecules of these schizophrenia risk genes. Targeting signaling molecules downstream of the histone-lysine N-methyltransferase *SETD1A*, one of the first identified schizophrenia risk genes, has shown promise, with cyclic AMP (cAMP)/protein kinase A (PKA) inhibitors rescuing network activity in mouse studies, and lysine-specific demethylase 1 (LSD1) inhibitors advancing into Phase II clinical trials for treating the negative and cognitive symptoms associated with schizophrenia [28,29]. *SP4* and *GRIN2A*, the schizophrenia risk genes identified as both common SNPs and rare SNVs, converge upon NMDAR as a critical determinant of schizophrenia disease pathophysiology, directing the development of novel therapeutics for schizophrenia towards targeting glutamatergic signaling and synaptic strength rather than dopaminergic signaling [47].

Ongoing clinical trials are evaluating drugs targeting glutamatergic signaling for the treatment of schizophrenia. Development of therapeutics targeting glutamatergic signaling has thus far focused on modifying the activity level of NMDARs through various mechanisms, including inhibition of endogenous NMDAR inhibitors, inhibition of NMDAR positive allosteric modulator (PAM) reuptake, activation of metabotropic glutamate receptors, and inhibition of voltage-gated sodium channels that enhance glutamate release [10]. While several of these approaches have shown promise in preclinical studies and early-phase clinical trials, their efficacy in producing consistent improvement in negative and cognitive symptoms remains to be fully established. This uncertainty suggests that altered glutamatergic signaling in schizophrenia may reflect dysregulation not only at the level of receptor activity, but also within the intracellular signaling mechanisms that regulate synaptic function and plasticity. Alterations in intracellular signaling components have been reported in schizophrenia, including changes in G-protein signaling observed in peripheral cells, although the mechanistic relationship of these findings to central synaptic dysfunction remains unresolved [48]. Targeting intracellular mechanisms that dynamically regulate NMDAR activity, trafficking, synaptic localization, as well as broader signaling pathways involved in synaptic plasticity, may provide a more effective means of restoring glutamatergic signaling and synaptic function. One class of intracellular signaling molecules broadly involved in signal amplification and feedback at the synapse is kinases, with kinases like Akt (*AKT1*), Pyk2 (*PTK2B*), protein kinase C β (*PRKCB*), and polo-like kinase (*PLK2*) identified as risk genes in schizophrenia [10]. Of these kinases, only Pyk2 directly regulates NMDAR activity, while Akt activity is regulated by NMDAR activity [30,49]. Phosphatases are integral components of these same feedback networks, acting not only to oppose kinase activity but to regulate the activity, trafficking, and localization of phosphorylation-dependent synaptic proteins. By modulating the activity of dysregulated kinases alongside other phosphorylation-dependent synaptic proteins, phosphatases represent a mechanistically compelling class of therapeutic targets for restoring synaptic signaling in schizophrenia.

## 3. Protein Phosphatases Implicated in Schizophrenia

**Figure 1:**
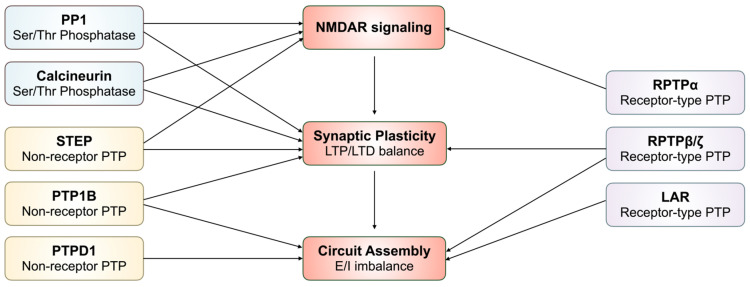
Schematic of how phosphates implicated in schizophrenia converge on shared hubs governing NMDAR signaling, synaptic plasticity, and circuit assembly.

Protein phosphatases play a central role in regulating synaptic signaling, plasticity, and neuronal circuit function by counterbalancing kinase-mediated phosphorylation events that control receptor activity, intracellular signaling cascades, and gene expression [50,51]. The human genome encodes four major protein phosphatase superfamilies: PTPs, STPs, histidine phosphatases, and haloacid dehalogenase (HAD) phosphatases. In the context of schizophrenia, converging genetic, post-mortem, and functional studies increasingly implicate dysregulated phosphatase activity as a contributor to the synaptic and circuit-level abnormalities underlying cognitive impairment, negative symptoms, and psychosis [52]. Multiple phosphatases, including both PTPs and STPs, have been linked to schizophrenia risk or pathology through alterations in expression or regulatory control, with particular relevance to glutamatergic neurotransmission, excitation–inhibition balance, and long-term synaptic plasticity (Table 2). This chapter focuses on protein phosphatases that have been most strongly implicated in schizophrenia, summarizing evidence from human genetic and brain studies as well as mechanistic insights from cellular and animal models, and highlighting how phosphatase dysregulation may converge on shared pathophysiological pathways despite the clinical heterogeneity of the disorder (Figure 1).

### 3.1. PP1 (PPP1C)

**Figure 2:**
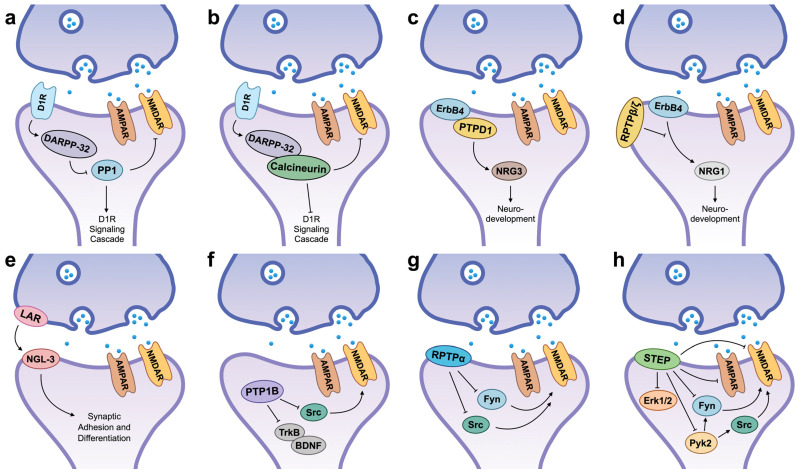
Protein phosphatases implicated in schizophrenia regulate synaptic signaling, plasticity, and neurodevelopmental pathways. Schematic overview of key serine/threonine and tyrosine phosphatases implicated in schizophrenia (SCZ) and their major synaptic signaling pathways. (**a**) Protein phosphatase 1 (PP1; PPP1C) is inhibited by DARPP-32 downstream of dopamine D1 receptor (D1R) signaling. (**b**) Calcineurin (PPP3C) counterbalances PP1 signaling and regulates dopamine- and NMDA receptor–dependent forms of synaptic plasticity. (**c**,**d**) PTPD1 (PTPN21) and RPTPβ/ζ (PTPRZ1) modulate ErbB4 signaling downstream of neuregulins (NRG3 and NRG1, respectively), influencing neurodevelopment. (**e**) LAR (PTPRF) regulates presynaptic differentiation and synaptic adhesion through trans-synaptic interactions with netrin-G ligand-3 (NGL-3). (**f**) PTP1B (PTPN1) negatively regulates BDNF/TrkB signaling and downstream Src family kinases, influencing long-term potentiation and synaptic strength. (**g**) RPTPα (PTPRA) dephosphorylates Src and Fyn kinases to regulate NMDA receptor phosphorylation. (**h**) STEP (PTPN5) directly dephosphorylates NMDA and AMPA receptor subunits as well as ERK1/2, Pyk2, and Fyn. Collectively, these phosphatases converge on glutamatergic signaling, excitation–inhibition balance, and activity-dependent synaptic plasticity—processes consistently disrupted in schizophrenia.

Protein phosphatase 1 (PP1; *PPP1C*) is an STP that regulates the activity of voltage-dependent sodium and calcium channels, cAMP-response element-binding protein (CREB), and neurotransmitter receptors in the brain (Figure 2a). Inhibition of PP1 by dopamine and adenosine 3′:5′-monophosphate-regulated phosphoprotein of 32 kDa (DARPP-32) potentiates dopaminergic signaling, increasing the activity of these ion channels and neurotransmitter receptors and regulating CREB signaling [17]. While altered PP1 expression or function has not yet been characterized in schizophrenia pathophysiology, DARPP-32 dysfunction has been suggested to contribute to the positive and negative symptoms of schizophrenia. Though genetic studies have shown mixed results for schizophrenia-associated DARPP-32 SNPs, multiple post-mortem studies have identified reduced DARPP-32 in the dorsolateral prefrontal cortex (DLPFC) of schizophrenic subjects compared to healthy controls. Interestingly, mRNA expression of DARPP-32 was found to be unchanged, suggesting that reduced DARPP-32 levels associated with schizophrenia are a result of translational or post-translational differences [53]. Though it is accepted that DARPP-32-mediated regulation of PP1 contributes to synaptic plasticity and cognitive function, more studies are needed to clarify how PP1 directly impacts synaptic function.

### 3.2. Calcineurin (PPP3C)

Calcineurin (*PPP3C*) is a calcium-dependent STP that is highly expressed in the central nervous system. Through its calcium-dependent activity, calcineurin contributes to regulation of dopaminergic signaling and certain forms of NMDAR-dependent synaptic plasticity (Figure 2b). Genetic association studies have determined that the catalytic subunit gamma of calcineurin (*PPP3CC*) maps to schizophrenia susceptibility loci. Forebrain-specific knockout of calcineurin’s regulatory subunit induced schizophrenia-like behavior in mice and disrupted the balance between LTD/LTP [18,30]. However, post-mortem studies show mixed results; notably, one study reported no difference in calcineurin protein levels in the DLPFC or hippocampus of schizophrenia patients compared with matched healthy controls [70]. Whether calcineurin hypofunction or altered expression underlies its contribution to schizophrenia-related phenotypes remains to be clarified.

### 3.3. PTPD1 (PTPN21)

PTPD1 (PTPD1; *PTPN21*) is a non-receptor type PTP that binds to V-erb-b2 avian erythroblastic leukemia viral oncogene homolog 4 (ErbB4), upregulating downstream signaling molecules like neurotrophic factor Neuregulin 3 (NRG3) and ultimately enhancing cortical neuron survival and neurite length [19] (Figure 2c). Based on GWAS analyses, two non-synonymous SNPs from *PTPN21* were associated with schizophrenia, marking it as a schizophrenia risk gene [54]. However, beyond its role in promoting neuronal survival and neurite elongation, the impact of altered *PTPN21* function on synaptic connectivity, circuit maturation, or neurotransmission relevant to schizophrenia remains unclear. Further studies examining *PTPN21* expression, regulation, and functional consequences in post-mortem samples and disease-relevant models are required to clarify its contribution to schizophrenia pathophysiology.

### 3.4. LAR (PTPRF)

Leukocyte common antigen-related phosphatase (LAR; *PTPRF*) is a receptor-type PTP that regulates the development of presynaptic glutamatergic terminals through modulating axon guidance and cytoskeletal organization [10,55,56] (Figure 2d). Through its trans-synaptic interaction with netrin-G ligand-3 (NGL-3), LAR regulates synaptic adhesion. *PTPRF* has been identified as a schizophrenia risk gene [10]. Loss-of-function mutations of *PTPRF* in Drosophila models resulted in disrupted axon guidance in neurons, while knockout of *Ptprf* in mice resulted in increased proliferation of neural precursor cells in the hippocampus, decreased hippocampal cholinergic innervation, decreased number and size of forebrain cholinergic neurons, decreased axonal regeneration, and behavioral hyperactivity and spatial learning impairments [55,57]. Despite this, post-mortem studies note no difference in *PTPRF* levels in the DLPFC of schizophrenia subjects compared with matched healthy controls and no correlation of *PTPRF* mRNA expression with any dendritic parameter [56]. Further analysis of *PTPRF* expression in established models of schizophrenia is needed to clarify the extent of *PTPRF*’s role in the pathophysiology of schizophrenia.

### 3.5. RPTPα (PTPRA)

Similarly, converging evidence points to another phosphatase, RPTPα, in which loss-of-function variants and knockout models demonstrate schizophrenia-related behavioral abnormalities and molecular alterations. RPTPα (*PTPRA*) is a receptor-type PTP that regulates the activity of kinases Src and Fyn, both of which are implicated in regulating NMDAR activity (Figure 2e). Like *PPP3RC*, *PTPRA* maps to psychotic illness susceptibility loci [20]. Further, whole-exome sequencing studies have identified rare missense variants of PTPRA associated with schizophrenia [58]. Knockout of *Ptpra* in mice not only induced schizophrenia-like behaviors but also reduced myelination, NMDAR phosphorylation, and LTP, paralleling observations of hypomyelination, NMDAR hypofunction, and decreased plasticity in schizophrenia. Post-mortem analysis of *PTPRA* mRNA expression in the DLPFC of schizophrenia patients compared with matched healthy controls revealed a significant decrease in expression in schizophrenia patients [20]. However, other association analyses did not find significant enrichment of rare *PTPRA* variants in schizophrenia, and the variants identified across studies are largely non-overlapping, highlighting substantial genetic heterogeneity [59]. Further studies are needed to functionally validate whether these rare and inconsistently associated variants meaningfully alter protein expression or function and clarify the molecular pathways through which RPTPα might influence schizophrenia pathophysiology.

### 3.6. RPTPβ/ζ (PTPRZ1)

RPTPβ/ζ (*PTPRZ1*) is a receptor-type PTP that inhibits neuregulin-1 (NRG1)-ERBB4 signaling, a pathway strongly implicated in schizophrenia pathophysiology through its role in neurodevelopment and synaptic plasticity (Figure 2f). Genetic studies have identified NRG1 as among the most replicated schizophrenia susceptibility genes, and a growing body of evidence implicates *ERBB4* and *PTPRZ1* as genetically associated with schizophrenia [21,60]. Mice overexpressing RPTPβ/ζ exhibited reduced NRG1-ERBB4 signaling accompanied by altered glutamatergic, GABAergic, and dopaminergic activity, and schizophrenia-like behaviors. Post-mortem analysis revealed significantly increased RPTPβ/ζ expression in the DLPFC of schizophrenia patients compared with matched healthy controls [21]. However, other genetic and rodent studies provide conflicting results. No genetic association was found between schizophrenia patients and *PTPRZ1* in either allelic, genotypic, or haplotypic analyses [61]. Like overexpression of *Ptprz*, genetic knockout of *Ptprz* in mice also increased dopamine levels in the PFC and hippocampus and schizophrenia-like behaviors [62]. Together these data suggest that the extent to which RPTPβ/ζ generalizes across patient populations and the directionality of its pathogenic mechanism in schizophrenia requires further clarification.

### 3.7. PTP1B (PTPN1)

PTP1B also demonstrates elevated activity associated with schizophrenia-like behaviors. PTP1B (*PTPN1*) is non-receptor PTP that primarily regulates metabolic and inflammatory signaling pathways in the liver, adipose tissue, and skeletal muscle, but also plays a role in the brain through modulating the brain-derived neurotrophic factor (BDNF)/tropomyosin receptor kinase B (TrkB) pathway implicated in LTP [22] (Figure 2g). Endogenous overactivation of PTP1B in mice induced schizophrenia-like behaviors, and subsequent genetic ablation or pharmacological inhibition of PTP1B ameliorated these behaviors [63]. However, the precise molecular pathways through which PTP1B contributes to schizophrenia pathophysiology remain unclear. Several studies propose that PTP1B may influence LTP through regulation of BDNF/TrkB signaling [22], metabolic hormones such as leptin and insulin [63], and even Src-dependent modulation of NMDAR function [22,64]; however, these mechanisms have not converged into a unified model.

### 3.8. STEP (PTPN5)

Striatal-enriched tyrosine phosphatase (STEP; PTPN5) is a neuron-specific PTP highly enriched in cortico-striatal circuits. In contrast to other phosphatases discussed, STEP directly regulates NMDAR and AMPAR function, as well as extracellular signal-regulated kinases (ERK1/2) and Fyn, positioning it as a central modulator of excitatory-inhibitory balance and synaptic plasticity (Figure 2h). Among the multiple STEP splice variants, STEP_46_ and STEP_61_ represent the major isoforms and exhibit distinct subcellular and regional distributions. STEP_46_ is primarily cytosolic, whereas STEP_61_ is membrane-associated due to an extended N-terminal sequence that targets it to intracellular membranes, including the postsynaptic density (PSD). While both isoforms are expressed in striatal and limbic regions, STEP_61_ is additionally present in the hippocampus and neocortex, suggesting a greater potential influence on cognitive processes. STEP_61_ has traditionally been described as enriched at the PSD of excitatory neurons but has also been detected in select interneuron populations and, more recently, at presynaptic membranes [65,71]. Consistent with their localization, STEP isoforms engage overlapping but distinct substrates. STEP_46_ preferentially dephosphorylates ERK1/2 and p38, whereas STEP_61_ targets synaptic substrates, including NMDAR and AMPAR subunits (GluN2B and GluA2), as well as Pyk2 and Fyn. Through ERK1/2 dephosphorylation, STEP_46_ opposes signaling pathways associated with LTP, biasing synapses toward LTD. STEP_61_-mediated dephosphorylation of Pyk2 and Fyn attenuates Src-family kinase signaling, thereby reducing NMDAR phosphorylation and activity. Direct dephosphorylation of GluN2B and GluA2 promotes clathrin-dependent receptor internalization, further shifting synaptic plasticity toward LTD [66].

STEP is tightly coupled to dopaminergic signaling. Activation of D1 receptors leads to STEP inactivation, stabilizing synaptic NMDARs and enhancing their activity [23]. This suggests that STEP may contribute to antipsychotic-associated modulation of glutamatergic signaling [24]. Indeed, chronic administration of typical or atypical antipsychotics results in STEP inactivation and increased surface expression of GluN1/GluN2B receptors in mice. Standard genetic and pharmacologically induced mouse models of schizophrenia show elevated STEP levels, and subsequent genetic ablation or pharmacological inhibition of STEP both ameliorated these behaviors, rescued NMDAR phosphorylation, and increased neuronal activity [67,68,72].

STEP also interacts with cholinergic signaling pathways. In hippocampal neurons, M1 AChR activation potentiates NMDAR currents, an effect enhanced by STEP inhibition. Notably, STEP inhibition or genetic deletion alone does not increase NMDAR currents, indicating that STEP functions as a regulator rather than a primary driver of excitatory tone. Mechanistically, calcium source appears to influence signaling downstream of M1 activation: calcium influx through NMDARs promotes Src activation, whereas intracellular calcium release favors STEP activation and NMDAR inactivation [73]. These dynamics may be relevant to schizophrenia, where NMDAR hypofunction predominates in prefrontal cortical circuits, while increased excitation is observed in corticolimbic circuits [10]. Elevated levels of STEP observed in schizophrenia may therefore reflect a maladaptive extension of a homeostatic mechanism that constrains excessive glutamatergic signaling in subcortical regions but exacerbates cortical NMDAR hypofunction. These findings suggest that therapeutic strategies targeting STEP may complement muscarinic receptor-based approaches by biasing M1 signaling toward NMDAR potentiation in cortical circuits while avoiding exacerbation of glutamatergic hyperactivity in subcortical regions.

Genetic association studies have provided preliminary evidence linking *PTPN5* to schizophrenia risk with nominally associated SNPs and haplotypes reaching false discovery rate (FDR)-corrected significance, particularly in males [23]. Post-mortem analysis of STEP has yielded mixed results: STEP protein levels in the DLPFC of unmedicated schizophrenia patients were increased [67], while STEP mRNA and protein levels in the DLPFC of medicated schizophrenia patients remained similar to matched healthy controls [69], suggesting potential sensitivity to treatment status. Notably, schizophrenia patient-derived human induced pluripotent stem cell (hiPSC) forebrain neurons from two cohorts exhibited elevated STEP_61_ expression overall, though this difference was driven by a subset of patients, indicating that elevated STEP levels may represent a disease-associated feature within specific patient subgroups [68]. Such disease heterogeneity highlights the importance of biomarker-guided stratification for phosphatase-targeted therapeutic strategies.

STEP expression has traditionally been characterized as postnatal. However, detection of functional STEP protein in embryonic rat neuronal cultures and patient-derived hiPSC-derived neurons suggests that STEP may operate during earlier developmental stages [67]. This finding is particularly significant because it suggests that elevated STEP levels may represent an early neurodevelopmental feature of schizophrenia pathophysiology rather than solely a consequence of disease progression or medication effects.

## 4. Challenges and Opportunities in Targeting Phosphatases for Schizophrenia Treatment

Despite growing evidence implicating protein phosphatases in the pathophysiology of schizophrenia, translating these findings into viable therapeutic strategies remains highly challenging. Within the PTP superfamily, which comprises more than 100 members in humans, a major obstacle is the highly conserved and positively charged active site, which often yields inhibitors with poor selectivity and limited cellular permeability [74,75,76,77,78]. Similarly, STPs such as PP1 and calcineurin possess highly conserved catalytic cores and derive substrate specificity primarily through association with regulatory subunits. Consequently, direct catalytic inhibition of phosphatases carries a substantial risk of off-target effects and disruption of essential neuronal functions. This section discusses recent advances in phosphatase inhibition relevant to schizophrenia, outlines key translational barriers to targeting these enzymes, and highlights emerging strategies aimed at achieving pathway- and circuit-specific modulation without globally suppressing phosphatase activity.

### 4.1. Inhibition of STEP and Restoration of Synaptic Receptor Trafficking

The STEP phosphatase has emerged as a key regulator of synaptic signaling and plasticity in the central nervous system. Multiple studies indicate that levels of active STEP are elevated not only in schizophrenia but also in Alzheimer’s disease (AD), Parkinson’s disease, and fragile X syndrome [67,72,79,80,81,82,83]. The prevailing model derived from these findings suggests that increased STEP activity interferes with synaptic function and contributes to the characteristic cognitive and behavioral deficits observed across these disorders [71]. Importantly, genetic knockout of STEP ameliorates biochemical, synaptic, and cognitive deficits in mouse models of schizophrenia, AD, and fragile X syndrome, thereby validating STEP as a potential target for drug discovery [67,72,82]. Notably, STEP knockout mice are viable, fertile, and appear phenotypically normal [84], suggesting that pharmacological inhibition of STEP may be well tolerated and could offer a disease-modifying therapeutic strategy for schizophrenia and other central nervous system (CNS) disorders.

**Figure 3:**
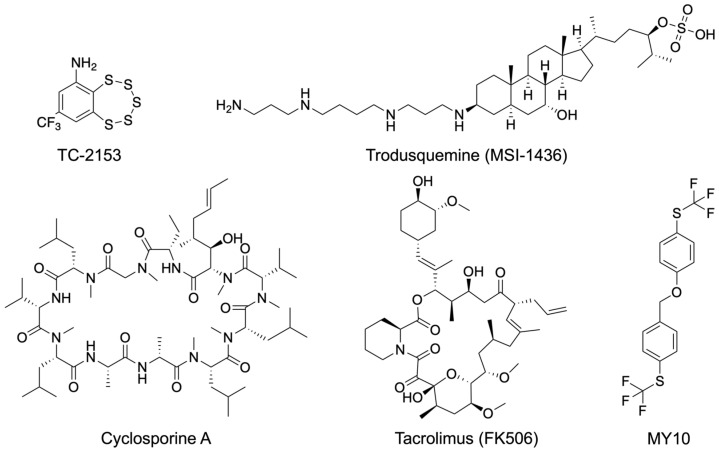
Schizophrenia-relevant phosphatase inhibitors. See text for details.

Efforts to identify STEP inhibitors led to the discovery of compound TC-2153 (Figure 3) [85]. TC-2153 selectively inhibited STEP over related PTPs in cellular and in vivo contexts. Although little selectivity was observed in biochemical assays using truncated phosphatase domains, substantial selectivity for STEP emerged when full-length enzymes were examined. These findings indicate that amino acid sequences outside the conserved catalytic domain contribute critically to inhibitor selectivity. Initial studies with TC-2153 showed that the compound effectively reversed the cognitive and cellular deficits in AD mice [85]. Subsequent work showed that STEP inhibition by TC-2153 increased phosphorylation of STEP substrates in both cellular and in vivo models of schizophrenia, including schizophrenia patient-derived hiPSC forebrain neurons (SZ1-FB) and Nrg1^+/−^ mice [68]. TC-2153 treatment also enhanced spontaneous neuronal activity in hiPSC-derived neurons and reversed behavioral and cognitive deficits in Nrg1^+/−^ mice. Despite these promising results, which further validated STEP as a drug target in schizophrenia and AD, TC-2153 is unlikely to advance as a therapeutic candidate. This limitation stems from the benzopentathiepin scaffold, which is known to react with cellular thiols, modify DNA, and exhibit cytotoxicity [31,86,87,88].

Additional efforts to develop STEP inhibitors have yielded phosphotyrosine (pTyr) substrate-mimicking phosphonic acids with good potency and moderate selectivity for STEP [89]. However, in our hands, the lead compound from this study failed to penetrate cell membranes, likely due to its highly charged chemical nature. Other reported STEP inhibitors include a series of quinazoline compounds with submicromolar potency [90,91]. To evaluate these candidates, we resynthesized the 16 most promising quinazolines and tested them against STEP and related phosphatases. None of the compounds exhibited appreciable selectivity for STEP, underscoring the persistent challenges associated with developing selective inhibitors for PTPs. In a more recent effort, a biophysical, label-free high-throughput screening (HTS) platform based on protein thermal shift (PTS) technology was established [92]. In contrast to conventional enzymatic HTS assays for STEP, which are prone to high false-positive rates, the PTS platform proved to be robust and capable of identifying true hits with confirmed STEP inhibitory activity and selectivity.

### 4.2. Allosteric Inhibition of PTP1B in Schizophrenia-Relevant Signaling Pathways

Pharmacological inhibition of PTP1B has emerged as a mechanistically grounded strategy for modulating signaling pathways relevant to schizophrenia. PTP1B is a non-receptor PTP that negatively regulates multiple receptor tyrosine kinases implicated in synaptic plasticity and cognition, including insulin receptors, leptin receptors, and BDNF/TrkB signaling. Foundational studies demonstrated that PTP1B directly associates with TrkB in the brain and acts as a physiological brake on BDNF signaling. Genetic deletion or pharmacological inhibition of PTP1B enhanced TrkB phosphorylation and downstream Akt and ERK signaling in vivo, establishing PTP1B as a key negative regulator of central neurotrophic signaling (extensively reviewed in Olloquequi et al., Ref. [22]).

Building on this mechanistic framework, subsequent inhibitor studies have linked excessive PTP1B activity to schizophrenia-like phenotypes in preclinical models. In mice exposed to subanesthetic ketamine, pharmacological inhibition of PTP1B using the allosteric inhibitor trodusquemine (MSI-1436) (Figure 3) reversed deficits in working memory, prepulse inhibition, and hyperlocomotion, behavioral domains commonly used as schizophrenia endophenotypes [64]. Similarly, in LMO4-deficient mice, an endogenous model characterized by elevated PTP1B activity and prefrontal cortical dysfunction, PTP1B inhibition restored TrkB-dependent endocannabinoid signaling, normalized Akt pathway activity, and rescued behavioral abnormalities [63,93]. At the synaptic level, PTP1B inhibition enhanced phosphorylation of signaling proteins linked to NMDAR function and long-term potentiation, consistent with improved synaptic plasticity [64].

Together, these studies indicate that PTP1B is pharmacologically tractable in the brain and that partial inhibition can enhance neurotrophic and metabolic signaling pathways converging on synaptic and circuit function. However, PTP1B also plays central roles in peripheral metabolic and inflammatory regulation, raising concerns about systemic effects associated with chronic inhibition. Thus, while PTP1B inhibition highlights an important opportunity to modulate convergent signaling mechanisms relevant to schizophrenia, achieving sufficient CNS selectivity and pathway specificity remains a key translational challenge.

### 4.3. Calcineurin Inhibition and CNS-Relevant Behavioral and Synaptic Effects

Calcineurin plays a central role in regulating glutamatergic synaptic plasticity, activity-dependent gene transcription, and cortical circuit stability. In neurons, calcineurin is a key molecular determinant of the balance between LTP and LTD, acting downstream of NMDA receptor-mediated calcium influx to promote LTD and depotentiation. Disruption of this balance, characterized by excessive LTD bias, impaired LTP, and NMDA receptor hypofunction, is a core feature of leading synaptic models of schizophrenia and has been implicated in cognitive deficits and negative symptoms.

Pharmacological studies using calcineurin inhibitors have provided insight into how perturbation of calcineurin activity influences schizophrenia-relevant behaviors and synaptic mechanisms. Systemic administration of cyclosporine A (CsA) alters prepulse inhibition (PPI) of the acoustic startle response in rodents, a well-established translational endophenotype of schizophrenia that reflects dysfunction in cortico-striatal–brainstem circuitry [94]. In addition, CsA has been shown to affect social and emotional behaviors and to modify dopamine and serotonin release in the PFC, a brain region central to executive dysfunction and cognitive control impairments in schizophrenia [95]. These findings indicate that calcineurin inhibition is sufficient to perturb behavioral and neurochemical domains directly relevant to psychosis and cortical dysfunction.

At the synaptic level, extensive electrophysiological and biochemical studies demonstrate that pan calcineurin inhibitors such as CsA and tacrolimus (FK506) (Figure 3) modulate NMDA receptor-dependent signaling and plasticity in cortical and hippocampal neurons. Inhibition of calcineurin disrupts LTD and depotentiation and alters NMDA receptor-mediated responses, consistent with a shift toward enhanced synaptic phosphorylation and potentiation [96,97]. These effects are mechanistically aligned with schizophrenia models in which excessive phosphatase activity is proposed to destabilize synapses and impair learning-related plasticity. Moreover, as highlighted in recent reviews, calcineurin inhibition can transiently restore phosphorylation of plasticity-associated proteins (e.g., CREB, CaMKII, AMPAR subunits) and improve cognitive performance under conditions of synaptic stress or pathological calcium signaling [98].

Despite these schizophrenia-relevant effects, both preclinical and clinical data underscore significant limitations of calcineurin inhibition as a therapeutic strategy. Calcineurin exerts broad control over neuronal excitability, synaptic scaling, and transcriptional homeostasis, and prolonged or global inhibition disrupts cognition, executive function, and emotional regulation. In clinical settings, long-term treatment with tacrolimus or cyclosporine A is frequently associated with cognitive impairment, mood disturbances, and psychotic symptoms, indicating that non-selective calcineurin inhibition can be neuropsychiatrically destabilizing [32,98]. These adverse effects are generally considered on-target consequences of widespread calcineurin suppression rather than off-target toxicity.

Collectively, CNS-focused inhibitor studies position calcineurin as a biologically important node in schizophrenia-relevant synaptic and circuit dysfunction, while simultaneously illustrating why direct catalytic inhibition is unlikely to be clinically viable. Instead, these findings support a model in which schizophrenia may involve context- or compartment-specific dysregulation of calcineurin signaling, and they highlight the need for therapeutic strategies that selectively modulate calcineurin-dependent pathways, such as downstream effectors or interacting signaling complexes, without globally suppressing calcineurin activity.

### 4.4. Pharmacological Inhibition of RPTPβ/ζ and Network-Level Signaling Effects

Pharmacological modulation of receptor-type PTPs such as *RPTPβ/ζ* has provided important insights into the feasibility and limitations of targeting phosphatases in the central nervous system. Recent inhibitor studies targeting RPTPβ/ζ) demonstrate that selective, brain-penetrant small molecules can engage phosphatase targets in vivo and induce biologically meaningful changes in CNS signaling and neuroimmune pathways. In the study by Fontán-Baselga et al., the small-molecule inhibitor MY10 (Figure 3), which binds the intracellular phosphatase domain of RPTPβ/ζ, was shown to reduce phosphatase activity and modulate downstream signaling cascades following oral administration in mice [99]. Pharmacological inhibition of RPTPβ/ζ altered the expression of genes involved in inflammatory signaling and proteostasis and reduced glial activation, indicating that partial phosphatase inhibition can reshape complex cellular networks in the adult brain without overt toxicity.

While these inhibitor studies were conducted in disease contexts other than schizophrenia, they nonetheless highlight both opportunities and challenges that are directly relevant to phosphatase targeting in schizophrenia. RPTPβ/ζ regulates kinase pathways, including Fyn- and Trk-dependent signaling, that intersect with glutamatergic neurotransmission, neurodevelopment, and glial-neuronal interactions, processes repeatedly implicated in schizophrenia pathophysiology. At the same time, the observed effects of RPTPβ/ζ inhibition reflect broad modulation of inflammatory and glial responses rather than selective correction of synaptic dysfunction, underscoring a key challenge for phosphatase-based therapies: disentangling disease-relevant signaling effects from pleiotropic network-level changes. Together with inhibitor studies targeting STEP and PTP1B, these findings suggest that phosphatases are pharmacologically tractable in the CNS, but that achieving pathway-, cell type-, and circuit-specific modulation remains a central barrier to translating phosphatase inhibition into effective schizophrenia therapeutics.

### 4.5. Novel Approaches Targeting Phosphatases for CNS Disorders

An important consideration in evaluating phosphatases as therapeutic targets is the inherent risk-benefit landscape associated with modulating enzymes that are frequently ubiquitous, multifunctional, and embedded within essential homeostatic networks. Unlike receptors, whose pharmacological manipulation can often be spatially restricted by expression patterns or ligand delivery, many phosphatases regulate signaling pathways across multiple tissues, raising the possibility of systemic liabilities when inhibition is not confined to the CNS. Achieving sufficient brain-selective exposure therefore represents a major translational challenge, particularly for targets such as PP1, calcineurin, or PTP1B, whose peripheral functions are tightly coupled to metabolic, immune, and cardiovascular regulation. Moreover, the highly conserved and charged active sites of protein phosphatases have historically constrained the development of selective, cell-permeable inhibitors. Collectively, these constraints suggest that broad catalytic-site inhibition may be neither feasible nor desirable for many phosphatases, as sustained global suppression risks disrupting physiological signaling balance. These considerations further suggest that successful therapeutic translation may require biomarker-guided strategies that identify patient subgroups in which phosphatase dysregulation contributes to disease pathophysiology. Such approaches could enable more targeted intervention while minimizing systemic liabilities associated with broadly modulating ubiquitous signaling enzymes.

**Figure 4:**
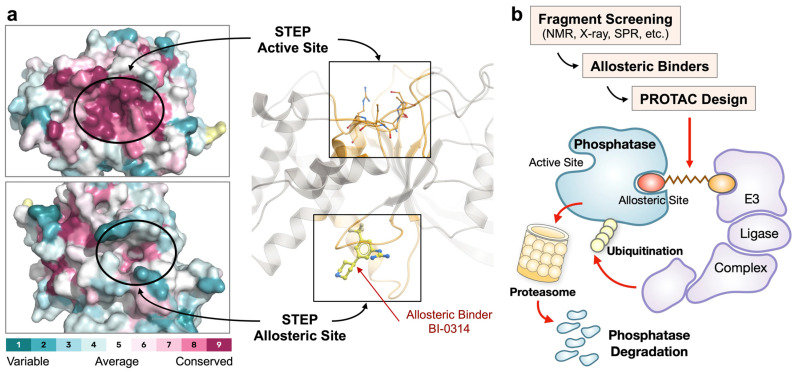
Challenges, opportunities, and emerging strategies for targeting protein phosphatases (**a**) Amino acid conservation across all 37 human classical phosphotyrosine-specific protein tyrosine phosphatases (PTPs). Protein sequences were aligned using Clustal Omega as implemented in MegAlign Pro (version 17; DNASTAR, Inc., Madison, WI, USA). Amino acid conservation scores were calculated and mapped onto the crystal structure of STEP (PDB ID: 6H8R) using the ConSurf Server (https://consurf.tau.ac.il, accessed on 1 September 2025). The protein surface, color-coded by amino acid conservation score, was visualized in PyMOL (version 3.1.6; Schrödinger, Inc., New York, NY, USA). The highly conserved active site and a druggable, non-conserved allosteric site within the STEP phosphatase are highlighted. A known STEP allosteric binder, BI-0314, is shown in stick representation (PDB ID: 68HS; Ref. [113]). (**b**) Non-conserved allosteric binding sites, as well as small molecule ligands targeting these sites, can be identified using fragment-based screening approaches. Fragment hits can be optimized into potent and selective binders that serve as starting points for the development of PROTACs, bifunctional molecules that recruit an E3 ubiquitin ligase complex, such as cereblon or VHL, promoting ubiquitination and subsequent degradation of the target phosphatase through the ubiquitin–proteasome system (UPS).

An emerging strategy to address these challenges involves targeting allosteric regulatory sites that are typically less conserved across phosphatase families (Figure 4a). Such approaches provide a plausible route to improved selectivity and tunable functional effects [100]. Allosteric pockets may also accommodate more drug-like chemical matter than catalytic sites, potentially improving physicochemical properties such as membrane permeability and blood–brain barrier (BBB) penetration [101]. Accordingly, allosteric inhibition of phosphatases has gained substantial traction [102,103,104], and the clinical feasibility of this approach has been demonstrated by the advancement of allosteric SHP2 inhibitors into clinical trials for cancer therapy [105]. Nevertheless, the allosteric mechanisms exploited by SHP2 inhibitors appear to be highly target-specific, and comparably effective allosteric inhibitors for most other phosphatases remain elusive.

Recent advances in drug discovery have further highlighted allosteric targeted protein degradation as a promising strategy to overcome long-standing challenges associated with drugging protein phosphatases [15] (Figure 4b). In contrast to conventional inhibitors that require engagement of conserved catalytic residues, degrader approaches can exploit non-orthosteric or regulatory surfaces to induce selective protein elimination. This paradigm is particularly well suited to phosphatases, whose disease relevance often reflects dysregulated localization, scaffolding, or complex assembly rather than simple gain or loss of catalytic activity. By enabling removal of both catalytic and non-catalytic functions, allosteric degraders offer a means to selectively reprogram phosphatase-driven signaling networks while minimizing the liabilities associated with direct catalytic inhibition.

Fragment-based drug discovery (FBDD) has emerged as a core enabling technology not only for identifying fragment hits as starting points for medicinal chemistry optimization, but also for uncovering novel druggable pockets, including cryptic binding sites, on the surface of target proteins [106]. In contrast to traditional HTS, which typically interrogates large libraries of chemically complex molecules and often yields hits that make multiple suboptimal interactions with the target protein, FBDD employs smaller libraries of low-complexity compounds that adhere to the “rule of three” (Ro3; molecular weight ≤ 300, cLogP ≤ 3, ≤3 hydrogen bond donors, and ≤3 hydrogen bond acceptors) [107]. Although fragment hits generally bind with lower affinity (high micromolar to low millimolar), they often engage targets through high-quality interactions. As a result, fragment hits typically exhibit higher ligand efficiencies (LEs), defined as the free energy of binding per heavy atom (LE = DG/N_HA_), an important metric for guiding hit prioritization and optimization [108,109,110].

Application of biophysical screening methods, including ligand-observed NMR spectroscopy, X-ray crystallography, microscale thermophoresis (MST), and differential scanning fluorometry (DSF; also referred to as protein thermal shift assays), has enabled the identification of allosteric binders and novel unique pockets in multiple human phosphatases, including PTP1B, STEP, VHR, and PTPN22 [111,112,113,114,115,116]. Importantly, non-inhibitory binders identified through such approaches can serve as valuable starting points for degrader-based therapeutics, particularly Proteolysis-targeting chimera (PROTAC) development, where target engagement rather than enzymatic inhibition is the primary requirement.

PROTACs represent the most advanced implementation of targeted protein degradation and have driven major advances in drug discovery by enabling selective elimination of previously intractable targets such as transcription factors and adaptor proteins [117]. PROTACs are heterobifunctional molecules composed of a ligand for the protein of interest (POI) linked to a ligand that recruits an E3 ubiquitin ligase, thereby promoting ubiquitination and proteasomal degradation of the POI [118]. Because PROTACs operate through an event-driven mechanism of action, they can achieve sustained target depletion at relatively low occupancy and are not limited to inhibition of enzymatic activity [119,120,121]. Importantly, PROTACs can be engineered to penetrate the BBB, and multiple CNS-directed degraders have demonstrated robust brain exposure and target engagement in preclinical models [122,123]. For example, aberrant tau has been successfully degraded in patient-derived neurons [124], and an orally bioavailable, BBB-penetrant PROTAC targeting leucine-rich repeat kinase 2 (LRRK2) has been reported [122]. In addition, Arvinas, Inc. (New Haven, CT, USA) is advancing several CNS-focused PROTAC programs, including LRRK2, mutant huntingtin, tau, and α-synuclein, toward clinical development, with preclinical data demonstrating effective BBB penetration and target degradation [125,126].

Taken together, these advances provide a compelling framework for revisiting protein phosphatases as therapeutic targets in CNS disorders. The combination of fragment-based discovery, biophysical screening, and targeted protein degradation offers a path to overcome the intrinsic challenges posed by conserved catalytic sites and complex regulatory architectures. Strategies that leverage allosteric binding sites and induce degradation offer the potential to selectively modulate phosphatase-driven signaling. As CNS-penetrant degraders continue to advance toward clinical application, these approaches may enable a new class of disease-modifying therapies targeting phosphatase-dependent mechanisms that have remained largely inaccessible to conventional pharmacology.

## 5. Conclusions

Schizophrenia remains a devastating neuropsychiatric disorder with inadequate treatment options, particularly for cognitive symptoms. It is increasingly understood as a disorder of disrupted synaptic signaling and circuit-level dysfunction rather than a consequence of isolated neurotransmitter abnormalities. While current antipsychotic treatments effectively reduce positive symptoms through dopaminergic modulation, they fail to adequately address the cognitive and negative symptom domains that most strongly determine long-term functional outcomes, including working memory, processing speed, and executive function. Clinical assessment of cognition encompasses multiple domains, including performance-based neuropsychological testing, interview-based functional assessments, and patient-reported cognitive difficulties, which do not always converge. Objective neuropsychological performance may be preserved while patients report a substantial subjective cognitive burden, or vice versa. Factors such as reduced insight into impairment and internalized stigma can further influence self-appraisal of cognitive deficits, highlighting that clinically meaningful cognitive improvement involves multiple dimensions of dysfunction rather than a single endpoint. Robust pharmacological treatments targeting the underlying synaptic pathophysiology are therefore needed to complement behavioral and psychosocial therapies. Genetic, post-mortem, and functional studies now converge on intracellular signaling pathways that regulate synaptic strength, plasticity, and excitatory-inhibitory balance as key drivers of disease pathophysiology. Within this framework, protein phosphatases emerge as central regulatory nodes that integrate calcium signaling, neuromodulatory input, and kinase activity to dynamically shape synaptic function.

Across multiple phosphatase families, convergent evidence implicates dysregulated phosphatase activity, rather than simple changes in expression, as a contributor to schizophrenia-relevant phenotypes. STPs such as PP1 and calcineurin influence synaptic plasticity through tightly regulated multiprotein complexes, while PTPs including PTP1B, RPTPα, RPTPβ/ζ, and STEP modulate receptor trafficking, neurotrophic signaling, and kinase-dependent synaptic pathways. Although these enzymes differ in localization, substrates, and developmental roles, a unifying theme is their impact on glutamatergic signaling and long-term synaptic plasticity. Importantly, pharmacological and genetic studies demonstrate that partial or context-dependent modulation of phosphatase activity can rescue schizophrenia-like behavioral and synaptic deficits in preclinical models, validating phosphatases as mechanistically relevant targets even when direct catalytic inhibition proves untenable.

Among the phosphatases discussed, STEP (*PTPN5*) stands out as a particularly compelling example of disease-relevant signaling dysregulation that may be amenable to therapeutic intervention. STEP is neuron-specific, directly targets NMDARs and AMPARs, and occupies a strategic position at the intersection of dopaminergic, cholinergic, and glutamatergic signaling. Elevated STEP activity has been observed across multiple schizophrenia models, patient-derived neurons, and post-mortem brain samples, and genetic or pharmacological reduction in STEP activity consistently rescues synaptic and behavioral phenotypes. Unlike broadly acting phosphatases such as calcineurin, STEP knockout mice are viable and phenotypically normal, suggesting a wider therapeutic window. These features collectively position STEP as a prototype for how selective modulation of phosphatase-driven synaptic mechanisms could complement existing treatments and address unmet cognitive symptoms.

Despite these advances, substantial challenges remain in translating phosphatase biology into effective schizophrenia therapies. The conserved architecture of phosphatase catalytic sites, extensive pleiotropy, and reliance on regulatory subunits have historically limited the development of selective, brain-penetrant inhibitors. However, emerging strategies, including allosteric modulation, fragment-based discovery, and targeted protein degradation, offer new opportunities to overcome these barriers. By enabling pathway-, cell type-, and circuit-specific control of phosphatase signaling, these approaches may allow selective reprogramming of synaptic dysfunction without globally suppressing essential enzymatic functions. As CNS-penetrant degraders and allosteric ligands continue to advance, phosphatases, long considered “undruggable,” may represent a new frontier for disease-modifying therapies that directly address the synaptic pathology underlying schizophrenia.

## Figures and Tables

**Table 2:** Protein phosphatases implicated in schizophrenia.

Phosphatase	Class	Primary Molecular Targets/Pathways	Mechanism Relevant to Synaptic Function	SCZ Genetic Evidence	SCZ Post-Mortem Evidence	SCZ Cellular/ hiPSC Evidence	SCZ Animal Model Evidence	Key Open Questions	Citations
PP1 (*PPP1C*)	STP	Ion channels, neurotransmitter receptors, CREB	Dephosphorylates ion channels and CREB; activity tightly controlled by DARPP-32	Mixed genetic evidence for DARPP-32 SNPs; PP1 itself not genetically implicated	Reduced DARPP-32 protein in DLPFC post-mortem	—	—	Whether PP1 activity itself is altered; DARPP-32 as indirect proxy	[17,53]
PTPD1 (*PTPN21*)	Non-receptor PTP	ErbB4/NRG3 signaling	Promotes cortical neuron survival and neurite outgrowth through ErbB4-dependent pathways	GWAS identified non-synonymous SNPs associated with schizophrenia	—	—	—	Functional role in synaptic dysfunction uncharacterized; behavioral effects unknown	[19,54]
LAR (*PTPRF*)	Receptor-type PTP	Axon guidance and synaptic adhesion machinery	Regulates presynaptic glutamatergic terminal development via cytoskeletal and trans-synaptic signaling (NGL-3)	Identified as schizophrenia risk gene	No change in post-mortem expression	—	Knockout induces schizophrenia-like developmental and behavioral phenotypes	Post-mortem expression unchanged despite genetic risk	[10,55,56,57]
Calcineurin (*PPP3C*)	STP	NMDAR-dependent plasticity, dopaminergic signaling	Couples calcium influx to LTD/LTP balance and dopamine-regulated synaptic signaling	PPP3CC maps to susceptibility loci	Mixed results (increased, de-creased, and no difference in levels found)	—	Forebrain knockout induces schizophrenia-like behavioral phenotypes	Post-mortem results mixed (medication status may stratify due to effect of D1R and D2R signaling)	[18]
RPTPα (*PTPRA*)	Receptor-type PTP	Src/Fyn kinases, NMDAR phosphorylation	Regulates NMDAR function indirectly by controlling Src family kinase activity	Rare missense variants; heterogeneous associations	Reduced DLPFC mRNA	—	Knockout induces schizophrenia-like behavioral and cellular phenotypes	Functional validation of rare variants needed; genetic heterogeneity unresolved	[20,58,59]
RPTPβ/ζ (*PTPRZ1*)	Receptor-type PTP	NRG1–ERBB4 signaling	Negatively regulates neurodevelopmental and synaptic plasticity pathways	No direct genetic association	Increased DLPFC expression post-mortem	—	Both overexpression and knockout produce schizophrenia-like behavioral and cellular phenotypes	Bidirectional animal findings unresolved; no genetic anchor; functional directionality unclear	[21,60,61,62]
PTP1B (*PTPN1*)	Non-receptor PTP	BDNF/TrkB, NMDAR-mediated plasticity, metabolic and inflammatory signaling	Modulates LTP and synaptic plasticity via neurotrophic and metabolic pathways	—	—	—	Overactivation induces schizophrenia-like behaviors; Inhibition rescues behavioral phenotypes	Genetic, post-mortem and cellular expression uncharacterized	[22,63,64]
STEP (*PTPN5*)	Non-receptor PTP	NMDARs, AMPARs, ERK1/2, Fyn, Pyk2	Directly dephosphorylates synaptic receptors and signaling kinases, biasing synapses toward LTD and reducing excitatory transmission	Some nominally associated SNPs and significant haplotypes	Increased levels in DLPFC (mixed results across studies)	Increased levels in patient-derived hiPSC neurons	Knockout or inhibition rescues schizophrenia-like behavioral and cellular phenotypes	Post-mortem inconsistency (medication status may stratify); no genetic link despite strong functional evidence	[23,65,66,67,68,69]

## Data Availability

No new data were created or analyzed in this study. Data sharing is not applicable to this article.

## Abbreviations

The following abbreviations are used in this manuscript:

AD	Alzheimer’s disease
AMPAR	α-amino-3-hydroxy-5-methyl-4-isoxazolepropionic acid receptor
BBB	blood–brain barrier
BDNF	brain-derived neurotrophic factor
CREB	cAMP-response element-binding protein
CNS	central nervous system
CsA	cyclosporine A
D1R	D1 receptor
D2R	D2 receptor
DARPP-32	adenosine 3′:5′-monophosphate-regulated phosphoprotein of 32 kDa
DLPFC	dorsolateral prefrontal cortex
DSF	differential scanning fluorometry
EPS	extra-pyramidal side effects
ErbB4	V-erb-b2 avian erythroblastic leukemia viral oncogene homolog 4
ERK1/2	extracellular signal-regulated kinases 1/2
FBDD	fragment-based drug discovery
GWAS	genome-wide association studies
HAD	haloacid dehalogenase
HTS	high-throughput screening
hiPSC	human induced pluripotent stem cell
KarXT	xanomeline-tropsium chloride
LAR	leukocyte common antigen-related
LRRK2	leucine-rich repeat kinase 2
LTD	long-term depression
LTP	long-term potentiation
LSD1	lysine-specific demethylase 1
mAChR	muscarinic acetylcholine receptor
MST	microscale thermophoresis
NGL-3	netrin-G ligand-3
NMDAR	N-methyl-D-aspartate receptor
NMR	nuclear magnetic resonance
NRG	neuregulin
PAM	positive allosteric modulator
PFC	prefrontal cortex
PKA	cyclic AMP (cAMP)/protein kinase A
POI	protein of interest
PP1	protein phosphatase 1
PPI	prepulse inhibition
PROTAC	proteolysis-targeting chimera
PSD	postsynaptic density
PTP	protein tyrosine phosphatase
PTS	protein thermal shift
pTyr	phosphotyrosine
QoL	quality of life
RPTP	receptor-type protein tyrosine phosphatase
SNP	single nucleotide polymorphism
SNV	single nucleotide variant
SPR	surface plasmon resonance
STEP	striatal-enriched tyrosine phosphatase
STP	serine/threonine phosphatase
TrkB	tropomyosin receptor kinase B
UPS	ubiquitin–proteasome system
